# Kinetic Profiles of Inflammatory Mediators in the Conjunctival Sac Fluid of Patients upon Photorefractive Keratectomy

**DOI:** 10.1155/2015/942948

**Published:** 2015-10-07

**Authors:** Veronica Tisato, Paolo Perri, Erika Rimondi, Elisabetta Melloni, Giuseppe Lamberti, Daniela Milani, Paola Secchiero, Giorgio Zauli

**Affiliations:** ^1^Department of Morphology, Surgery and Experimental Medicine and LTTA Centre, University of Ferrara, Via Fossato di Mortara 66, 44121 Ferrara, Italy; ^2^Department of Biomedical Sciences and Surgical Specialities, University-Hospital of Ferrara, Corso Giovecca 203, 44121 Ferrara, Italy; ^3^Department of Life Sciences, University of Trieste, Via Manzoni 16, 34137 Trieste, Italy; ^4^Institute for Maternal and Child Health, IRCCS Burlo Garofolo, Via dell'Istria 65/01, 34137 Trieste, Italy

## Abstract

Photorefractive keratectomy (PRK) represents a therapeutic option to remodel corneal stroma and to compensate refractive errors, which involves inflammatory and/or regenerative processes. In this context, the modulation of cytokines/chemokines in the conjunctival sac fluid and their role in the maintenance of the corneal microenvironment during the healing process upon refractive procedures has not been deeply investigated. In this study, serial samples of conjunctival sac fluid of patients (*n* = 25) undergoing PRK were harvested before and at different time points after surgery. The levels of 29 cytokines/chemokines/growth factors involved in inflammatory/immune processes were measured with a multiplex array system. The results have firstly highlighted the different pattern of cytokine expression between the microenvironment at the anterior surface of the eye and the systemic circulation. More importantly, the kinetic of modulation of cytokines/chemokines at the conjunctival level following PRK revealed that while the majority of cytokines/chemokines showed a significant decrease, MCP-1 emerged in light of its pronounced and significant increase soon after PRK and during the follow-up. This methodological approach has highlighted the role of MCP-1 in the healing process following PRK and has shown a potential for the identification of expression/modulation of soluble factors for biomarker profiling in ocular surface diseases.

## 1. Introduction

The generation and preservation of the microenvironment of the tear film is guaranteed by the biological interplay between the ocular surface together with the related glands and local neural interconnections, that represent the core players for the protection of the transparency of the cornea and the health of the ocular surface [1]. In particular, the tear layer can be considered a* reservoir* of soluble factors with biological effects involved in the maintenance of the physiology of surface epithelial cells including protective and antimicrobial activities, nourishing functions, and contribution to local wound healing/anti-inflammatory responses [2–4]. In normal conditions, it has been demonstrated that tears of healthy subjects are characterized by a so-called “immune tone” generated by local levels of cytokines and chemokines released by different cell types, such as immune cells and epithelial cells [5, 6]. Therefore, any alteration of the tear film may have direct consequences on the ocular surface integrity leading to pathological condition such as dry eye [7, 8]. However, at the same time, any changes on the composition of the conjunctival sac fluid might have a potential as clinical biomarker to monitor the onset-evolution of ocular surface diseases [9–11] and the response to pharmacological and/or surgical interventions [12–14].

Photorefractive keratectomy (PRK) is one of the most commonly used surgical techniques to correct refractive errors through a laser-induction ablation of the corneal epithelium, which is able to induce corneal stromal remodelling with changes in corneal refraction [15]. This procedure is followed by the release of several factors including cytokines, growth factors, and matrix metalloproteases that are involved in both corneal wound healing process and possible postoperative (inflammatory) complications [16]. In this light, PRK might be considered a model of corneal regeneration for studies aiming at improving the comprehension of the complex biological processes underlying the maintenance of local corneal microenvironment and the wound healing process.

Therefore, the objective of this study was to monitor the local cytokines/chemokines levels in patients undergoing PRK including a wide inflammatory/immune mediators' profile. By this experimental approach, we aimed (i) to increase the understanding of the physiopathological response after PRK in a long term postoperative period; (ii) to identify molecular mediators involved in the wound healing process after PRK; and (iii) to assess a methodological approach for the identification of molecular mediators that could act as laboratory biomarkers for prognostic and monitoring purposes in the context of ocular surface diseases.

## 2. Materials and Methods

### 2.1. Patients' Population and Conjunctival Sac Fluid Collection

The subjects involved in the study included 25 patients enrolled by the Ophthalmology Section at the University-Hospital of Ferrara. All patients underwent complete preoperatively ophthalmic examination before receiving myopic PRK treatment by using the 200 Hz Allegretto laser platform (Wavelight Laser Technologie AG, Erlangen, Germany). In particular, preoperative (1 day before PRK), as well as postoperative (2, 5, and 30 days after PRK), follow-up examinations included analyses of uncorrected distance visual acuity (UDVA), corrected distance visual acuity (CDVA), manifest refraction, corneal topography, and complications. Before and after PRK, at the time of patients' clinical assessment, conjunctival sac fluid samples were collected by using standard strips of Schirmer test as previously described [17]. Briefly, for each subject a volume corresponding to three notches of the strip was collected. Strips were then transferred in 400 *μ*L of 0.9% NaCl solution at 4°C for 36 hours, to allow the release of the conjunctival sac fluid proteins from the strip in the solution. As control, conjunctival sac fluid samples were also collected from the normal contralateral eye of the 52% of enrolled patients. Aliquots of conjunctival sac fluid solutions were stored at −80°C and thawed only once before analyses. Forearm blood samples were collected from healthy subjects in the presence of sodium citrate and immediately centrifuged for plasma isolation that were stored at −80°C in single-use aliquots. Written informed consents were obtained from each patient and all the procedures that followed were in accordance with the Declaration of Helsinki and were approved by the institutional review board (University Hospital of Ferrara).

### 2.2. Analysis of Cytokines and Chemokines

The biological samples were frozen and thawed only once before performing the MILLIPLEX MAP Human Cytokine/Chemokine Panel (Merck Millipore, Billerica, MA), a bead-based multiplex immunoassay, which allows the simultaneous quantification of the following 29 human cytokines: EGF, IL-1*β*, IL-1 receptor antagonist (RA), IL-1*α*, IL-2, IL-3, IL-4, IL-5, IL-6, IL-7, IL-8, IL-10, IL-12(p40), IL-12(p70), IL-13, IL-15, 1L-17A, EOTAXIN, G-CSF, GM-CSF, IFN-*α*2, IFN-*γ*, CXCL10, MCP-1, MIP-1*α*, MIP-1*β*, TNF-*α*, TNF-*β*, and VEGF. Samples were analysed in duplicate following the manufacturer's recommended protocols and the results were read on a MAGPIX instrument equipped with the MILLIPLEX-Analyst Software using a five-parameter nonlinear regression formula to compute sample concentrations from the standard curves, as previously described [18].

### 2.3. Network Analysis

The identified proteins were analysed by using the IPA software (http://www.ingenuity.com/; Ingenuity Systems, Redwood City, CA, USA) in order to identify biological functions and molecular pathways associating the proteins, as well as predict protein-protein interaction networks determined by the Ingenuity Knowledge Base [19]. Briefly, the Ingenuity Pathway Knowledge Base identifies protein networks that are ranked in relation to the biological functions assigned to the network. The score indicates the probability that eligible proteins are in the network by random chance. In other words, high-score values indicate high reliability of protein association (a score > 2 is considered to be significant). The most highly scored networks identified were then graphically visualized showing the major molecular relationship between proteins.

### 2.4. Statistical Analysis

Data were analysed by SPSS statistical software and calculated as median, mean ± standard deviation (SD) for each group of data obtained from samples analysis. Box plots were used to show the median values and 25th to 75th percentiles. Differences between values were evaluated by using a pairwise sign-rank Wilcoxon's test and a *p* value < 0.05 was considered statistically significant.

## 3. Results

### 3.1. Study Population and Clinical Assessment

The cohort of patients enrolled for this study consisted of 64% of females with age ranging between 24 and 48 years and a mean age of 36.1 years and of 36% of males characterized by age ranging between 22 and 54 years with a mean age of 36.7 years. After PRK treatment the postoperative course was clinically monitored: no complications such as keratitis, infections and/or delay on corneal epithelium regeneration were reported and all patients showed full correction of the refractive errors and restoration of a proper visual quality, with the exception of one patient showing hypermetropy at the last clinical follow-up assessment performed 30 days after surgical intervention.

### 3.2. Expression Profile of Cytokines and Chemokines in the Conjunctival Sac Fluid

**Figure 1:**
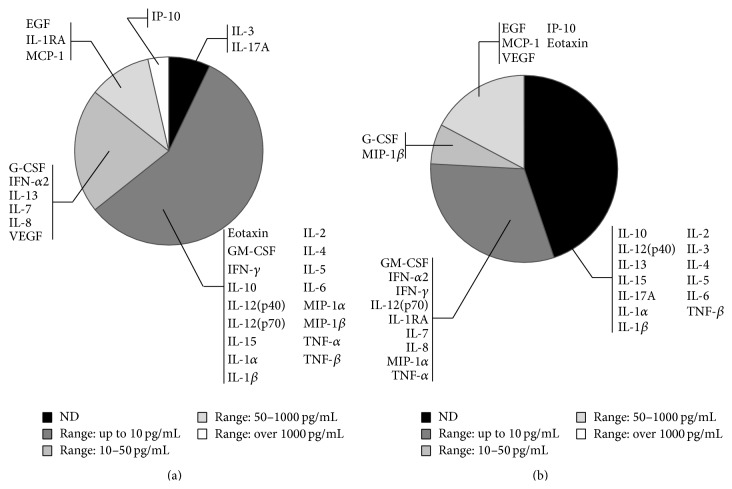
Comparison between local (microenvironment of the anterior surface of the eye) and systemic levels of cytokines/chemokines in healthy subjects. The levels of 29 cytokines/chemokines were measured by multiples assays in conjunctival sac fluid samples collected from normal contralateral eyes of patients (normal controls) (a) and serum samples (b) of healthy subjects. The levels of expression have been arbitrarily divided in ranges, as detailed in the legend, and graphically represented based on the number of cytokines/chemokines belonging to the different clusters. ND: not detectable.

In a preliminary experiment, we have analysed the levels of expression of 29 cytokines/chemokines in the conjunctival sac fluid samples collected from the normal contralateral eyes of patients (see Figure 1(a) and Supplementary Table 1 in Supplementary Material available online at http://dx.doi.org/10.1155/2015/942948). In particular, as summarized in Figure 1(a), among the 29 cytokines/chemokines analysed by multiplex assay, only IL-3 and IL-17A were undetectable in all samples while the following 27 cytokines/chemokines were detectable at different levels (up to over 1000 pg/mL): EGF, IL-1RA, MCP-1, IP-10/CXCL10, Eotaxin, GM-CSF, IFN-*γ*, IL-10, IL-12(p40), IL-12(p70), IL-15, IL-1*α*, IL-1*β*, IL-2, IL-4, IL-5, IL-6, MIP-1*α*, MIP-1*β*, TNF-*α*, TNF-*β*, IFN-*α*2, G-CSF, IL-13, IL-7, IL-8, and VEGF. Notably, the expression profile and the levels assessed in the conjunctival sac fluids were significantly different from those documented in serum samples of healthy subjects (Figure 1(b) and Supplementary Table 1). In fact, a higher number of soluble factors were undetectable at the serum level: IL-10, IL-12(p40), IL-15, IL-13, IL-17A, IL-1*α*, IL-1*β*, IL-2, IL-3, IL-4, IL-5, IL-6, and TNF-*β* (Figure 1(b)). Moreover, when clustered for ranges of expression levels, a limited number of cytokines/chemokines were detected at serum levels higher than 50 pg/mL and no soluble factor showed systemic levels over 1000 pg/mL (Figures 1(a)-1(b)).

### 3.3. Differential Modulation of Cytokines/Chemokines in the Conjunctival Sac Fluid following PRK Treatment

The patients enrolled in the study underwent PRK surgery and the levels of the same 29 cytokines/chemokines were evaluated in serial samples of conjunctival sac fluid collected one day before and at different time points (days 2, 5, and 30) after PRK. The levels of the cytokines and chemokines assessed before and after the PRK surgery are reported in Table 1. In order to identify clusters of cytokines/chemokines that might potentially be linked and interact at the conjunctival microenvironment level, we have firstly analysed the kinetics of release of the different cytokines/chemokines (Table 1). Then, in order to understand the potential association among the cytokines/chemokines having in common the same kinetic profile, the data were analysed using Ingenuity Pathway Analysis (IPA) software (http://www.ingenuity.com/) which identifies relevant interactions and biological mechanisms among proteins (Supplementary Table 2).

**Figure 2:**
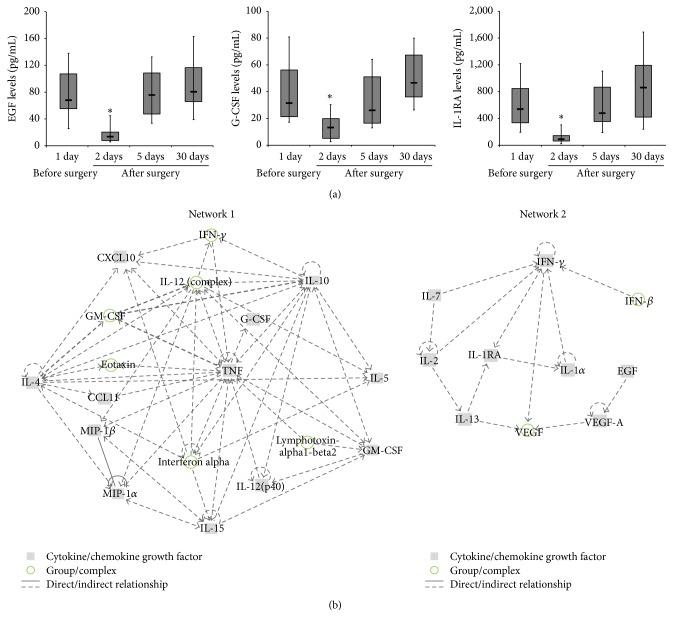
Characterization of Profile I kinetics of modulation of cytokines/chemokines in the conjunctival sac fluid of patients undergoing PRK treatment. The local levels of cytokines/chemokines were measured in conjunctival sac fluid samples collected from patients before and at the indicated time points after PRK. In (a), modulation of EGF, G-CSF, and IL-1RA is shown as representative of Profile I. Horizontal bars are median, upper and lower edges of box are 75th and 25th percentiles, and lines extending from box are 10th and 90th percentiles. Asterisks, ^*∗*^ *p* < 0.001 compared to the values before PRK. In (b), the identified panel of cytokines/chemokines sharing Profile I kinetics of modulation was assessed for network analysis. The two most highly scored networks are shown. The links between the different proteins generated by IPA (ingenuity pathway analysis) are graphically illustrated and the key for the nodes and the lines are reported on the legend.

Based on these analyses, we have identified two different kinetics of modulation, referred to as Profile I and Profile II. In particular, as detailed in Table 1, the majority of the cytokines/chemokines analysed, including EGF, IP-10/CXCL10, Eotaxin, GM-CSF, IFN-*γ*, IL-10, IL-12(p40), IL-12(p70), IL-15, G-CSF, IL-1*α*, IL-2, IL-4, IL-5, MIP-1*α*, MIP-1*β*, TNF-*α*, TNF-*β*, IFN-*α*2, IL-1RA, IL-13, IL-7, and VEGF, showed a dramatic decrease at day 2 after PRK, followed by a rapid recover at day 5 and thereafter (Profile I). The kinetics of modulation of EGF, G-CSF, and IL-1RA are shown in Figure 2(a) as representative of Profile I. Within the 23 cytokines/chemokines of Profile I, two networks were found with score >2 and involving 12 and 10 proteins, respectively (Figure 2(b)). Of interest, both of these networks involve a high number of focus molecules mainly related to immune cell trafficking, cellular growth/proliferation, and inflammatory responses (Supplementary Table 2).

**Figure 3:**
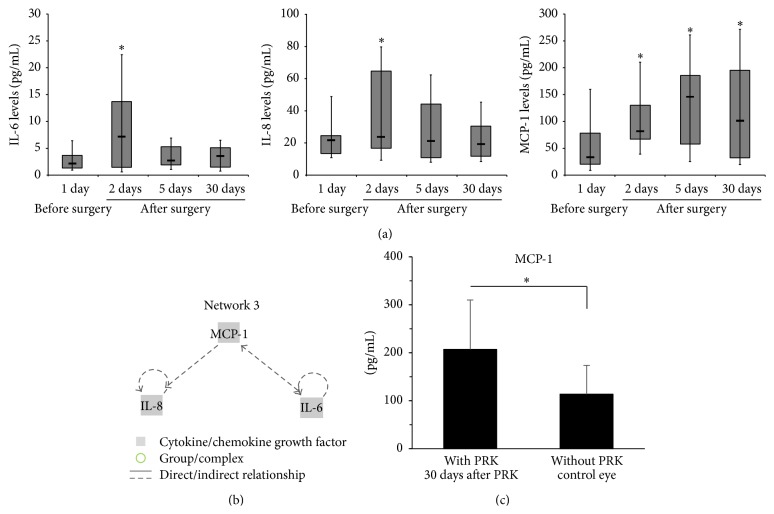
Characterization of Profile II kinetic of modulation of cytokines/chemokines in the conjunctival sac fluid of patients undergoing PRK treatment. The local levels of cytokines/chemokines were measured in conjunctival sac fluid samples collected from patients before and at the indicated time points after PRK. In (a), modulation of IL-6/8 and MCP-1 is shown. Horizontal bars are median, upper and lower edges of box are 75th and 25th percentiles, and lines extending from box are 10th and 90th percentiles. Asterisk, *p* < 0.05 compared to the values before PRK. In (b), the identified panel of cytokines/chemokines sharing Profile II was assessed for network analysis and the resulting networks are shown. The links between the different proteins generated by IPA (ingenuity pathway analysis) are graphically illustrated and the key for the nodes and the lines are reported on the figure legend. In (c), the levels of MCP-1 were assessed in the conjunctival sac fluid of patients 30 days after PRK and in their contralateral normal control eye. Results are reported as means ± SD, ^*∗*^ *p* < 0.05.

On the other hand, IL-6, IL-8, and MCP-1 showed a different profile of expression (Profile II) with respect to the majority of the cytokines/chemokines analysed, characterized by an increase at day 2 after PRK (Table 1 and Figure 3(a)). These proteins are functionally involved in a network related to inflammatory processes (Figure 3(b)). Anyhow, among the panel of 29 cytokines/chemokines assessed in the present study, the peculiar kinetic of expression/modulation of MCP-1 characterized by a significant (*p* < 0.05) and persistent increase at all times (up to 30 days) after PRK (Figure 3(a)) is certainly of note. Moreover, analysis of patient's matched samples shows that the levels of MCP-1 measured at the last clinical follow-up assessment performed 30 days after PRK were significantly (*p* < 0.05) different from the levels of MCP-1 detected in paired normal contralateral eyes (Figure 3(c)).

## 4. Discussion

The first observation derived from our study is that the profile of expression of cytokines/chemokines at the conjunctival sac fluid level does not match the systemic profile for the great majority of the cytokines/chemokines analysed in line with previous observations [11]. In this respect, we are aware that the procedures for the measurements of the cytokines and chemokines in tears show some difficulties and that the procedure employed to measure soluble cytokines/chemokines in conjunctival sac fluid might underestimate the real concentrations (since a percentage of the proteins could remain trapped in Schirmer strips). In spite of these potential technical limitations, the comparative analysis of the concentrations of the same cytokines/chemokines between serum samples and tears shows a more complex pattern of expression and higher levels of cytokines/chemokines in the microenvironment of the anterior surface of the eye, with respect to the systemic circulation. This observation is likely the results of the synthesis/release from different cellular source, such as epithelial cells and immune cells, which might contribute to generate the observed differences between serum and tears.

Moreover, we report for the first time the pattern of expression/modulation of a wide panel of cytokines/chemokines in the conjunctival sac fluid of patients before and after PRK treatment. As observed in a previous study focused on the analysis of a single cytokine (TRAIL) [17], involved in migration [20, 21] and inflammatory [22] processes, we found that the large majority of the cytokines/chemokines investigated in this study were characterized by a drastic decrease at day 2 after surgery, followed by a recover thereafter. Interestingly, only three cytokines (MCP-1, IL-6, and IL-8) did not show any significant decrease after surgery, with MCP-1 exhibiting the most remarkable pattern. Indeed, the MCP-1 levels in the conjunctival sac fluid showed a pronounced and significant increase already at day 2, peaking at day 5 after surgery and maintaining high levels up to day 30 after surgery. This pattern is particularly noteworthy, as MCP-1 has been involved in wound healing processes [23–27]. Therefore, it is likely that MCP-1 elevation plays a significant role in the process of reepithelialization of the cornea.

In conclusion, in the context of PRK, our data contribute to identify the key inflammatory/immune mediators involved in the local physiopathological responses to the surgical procedure and allowed to identify MCP-1 as a key molecular mediator involved in the wound healing process after PRK. Moreover, the present study, based on the application of a multiplex-array approach in the PRK model of corneal regeneration, certainly contributes to the improvements in the development of biomarker profiles for other ocular surface diseases requiring monitoring of the pathology development as well as monitoring after surgery.

## Supplementary Material

Supplementary Table 1. The levels of a panel of 29 cytokines/chemokines were measured by using a bead-based multiplex immunoassay in samples of control conjunctival sac fluids and peripheral blood.
Supplementary Table 2. Ingenuity Pathway analysis performed on the two described kinetic profiles of modulation of cytokines/chemokines in the conjunctival sac fluid of patients undergoing PRK has allowed the identification of three molecular Networks linking the molecules within the same kinetic profile.

## Figures and Tables

**Table 1:** Cytokines and chemokines levels in conjunctival sac fluid of patients undergoing PRK.

Cytokines and chemokines		1 day before PRK (pg/mL)^a^	Follow-up after PRK		
			2 days (pg/mL)^a^	5 days (pg/mL)^a^	30 days (pg/mL)^a^
EGF	Epidermal growth factor	68.7 (82.4 ± 52.3)	13.8 (25.0 ± 38.7)	76.7 (88.7 ± 65.6)	80.2 (95.2 ± 58.0)
Eotaxin	C-C motif chemokine ligand 11	0.0 (2.4 ± 4.8)	0.0 (0.8 ± 2.8)	6.4 (6.7 ± 7.0)	8.5 (7.1 ± 5.8)
G-CSF	Granulocyte colony-stimulating factor	31.5 (41.3 ± 27.0)	12.8 (15.4 ± 16.2)	26.1 (32.9 ± 24.4)	46.8 (52.7 ± 27.4)
GM-CSF	Granulocyte-macrophage colony-stimulating factor	0.7 (0.9 ± 0.7)	0.0 (0.4 ± 0.6)	1.1 (1.4 ± 0.8)	1.6 (1.4 ± 0.6)
IFN-*α*2	Interferon, alpha2	38.2 (47.0 ± 26.7)	23.7 (27.1 ± 19.9)	33.3 (33.7 ± 19.1)	44.4 (46.9 ± 24.6)
IFN-*γ*	Interferon, gamma	2.1 (3.3 ± 4.6)	0.0 (0.8 ± 0.9)	2.6 (2.8 ± 1.5)	2.7 (2.8 ± 1.2)
IL-10	Interleukin 10	1.3 (1.8 ± 1.0)	0.8 (0.7 ± 0.7)	1.6 (1.8 ± 1.1)	2.2 (2.3 ± 0.9)
IL-12(p40)	Interleukin 12 subunit p40	3.6 (3.3 ± 2.7)	2.8 (2.1 ± 2.3)	4.5 (4.1 ± 3.1)	5.1 (5.1 ± 3.6)
IL-12(p70)	Interleukin 12 complex	2.5 (2.5 ± 2.1)	0.0 (0.6 ± 1.2)	3.4 (3.2 ± 2.3)	3.9 (3.6 ± 2.2)
IL-13	Interleukin 13	14.3 (19.2 ± 14.1)	3.0 (5.2 ± 8.0)	14.6 (17.6 ± 13.3)	26.9 (29.9 ± 17.0)
IL-15	Interleukin 15	1.3 (1.2 ± 1.1)	0.0 (0.4 ± 0.7)	2.1 (1.9 ± 1.4)	2.1 (2.2 ± 1.2)
IL-17A	Interleukin 17	OOR<	OOR<	OOR<	OOR<
IL-1RA	Interleukin 1 receptor antagonist	562.0 (703.7 ± 562.5)	95.1 (141.5 ± 152.9)	498.0 (663.8 ± 579.5)	895.3 (920.4 ± 550.8)
IL-1*α*	Interleukin 1, alpha	11.0 (11.2 ± 5.1)	6.7 (8.4 ± 6.0)	13.5 (14.8 ± 6.5)	16.9 (19.0 ± 8.2)
IL-1*β*	Interleukin 1, beta	0.7 (0.7 ± 0.8)	0.7 (0.8 ± 1.0)	1.4 (1.4 ± 1.0)	1.6 (1.9 ± 1.1)
IL-2	Interleukin 2	0.0 (0.5 ± 0.7)	0.0 (0.2 ± 0.4)	0.7 (0.7 ± 0.6)	0.9 (0.9 ± 0.6)
IL-3	Interleukin 3	OOR<	OOR<	OOR<	OOR<
IL-4	Interleukin 4	5.25 (16.1 ± 36.7)	0.0 (4.7 ± 10.83)	3.6 (8.1 ± 15.3)	8.7 (12.1 ± 8.8)
IL-5	Interleukin 5	1.2 (2.7 ± 3.0)	0.0 (0.4 ± 1.3)	1.02 (1.9 ± 2.4)	2.4 (3.4 ± 3.2)
IL-6	Interleukin 6	2.4 (3.1 ± 2.4)	7.5 (9.9 ± 10.4)	2.9 (4.6 ± 5.5)	3.1 (3.5 ± 3.3)
IL-7	Interleukin 7	13.7 (16.1 ± 7.8)	4.9 (7.1 ± 8.6)	13.7 (16.9 ± 11.3)	24.8 (24.6 ± 11.0)
IL-8	Interleukin 8	22.3 (29.9 ± 18.2)	24.5 (38.8 ± 30.9)	21.3 (38.4 ± 35.2)	20.3 (30.2 ± 18.2)
IP-10	CXCL10	1432.0 (2026.6 ± 1896.5)	185.0 (435.0 ± 538.8)	2428.0 (3241.1 ± 2564.3)	2856.5 (3303.1 ± 1751.1)
MCP-1	CCL2	34.9 (83.4 ± 103.3)	80.0 (138.9 ± 104.1)	150.9 (144.2 ± 130.0)	115.9 (162.7 ± 128.5)
MIP-1*α*	CCL3	0.0 (2.3 ± 3.5)	0.0 (1.1 ± 2.1)	5.2 (4.0 ± 4.3)	5.2 (5.0 ± 4.4)
MIP-1*β*	CCL4	3.6 (4.5 ± 2.7)	1.3 (1.7 ± 1.8)	6.2 (5.7 ± 3.7)	5.6 (6.3 ± 3.2)
TNF-*α*	Tumor necrosis factor, alpha	0.0 (0.3 ± 0.6)	0.0 (0.0 ± 0.1)	0.7 (0.9 ± 1.3)	0.8 (0.7 ± 0.5)
TNF-*β*	Tumor necrosis factor, beta	0.0 (0.4 ± 0.8)	0.0 (0.1 ± 0.4)	0.0 (0.6 ± 0.9)	0.0 (0.7 ± 0.9)
VEGF	Vascular endothelial growth factor	29.1 (31.9 ± 17.4)	8.7 (12.2 ± 11.7)	29.1 (42.8 ± 50.9)	52.6 (52.8 ± 21.8)

^a^Values are expressed as median (mean ± SD); OOR<: out (below) of detection range.
