# Supplementary material for: Kinetic Profiles of Inflammatory Mediators in the Conjunctival Sac Fluid of Patients upon Photorefractive Keratectomy
Source: Mediators Inflamm. 2015 Oct 7;2015:942948. doi: 10.1155/2015/942948 (PMC4617429; doi:10.1155/2015/942948)
Supplement: Supplementary file 1 — Supplementary Table 1. The levels of a panel of 29 cytokines/chemokines were measured by using a bead-based multiplex immunoassay in samples of control conjunctival sac fluids and peripheral blood. Supplementary Table 2. Ingenuity Pathway analysis performed on the two described kinetic profiles of modulation of cytokines/chemokines in the conjunctival sac fluid of patients undergoing PRK has allowed the identification of three molecular Networks linking the molecules within the same kinetic profile. [file 942948.f1.pdf]

Supplementary Table 1. Cytokines and chemokines levels in control conjunctival sac fluids and blood samples

Cytokines and chemokines		Conjunctival sac fluid levels (pg/ml) [a]	Serum levels (pg/ml) [a]
**EGF**	Endothelial growth factor	105.0 (125.8±72.9)	242 (301.5±147.7)
**Eotaxin**	C-C motif chemokine 11; eosinophil chemotactic protein	4.4 (6.3±5.1)	85.7 (92.9±45.8)
**G-CSF**	Granulocyte colony-stimulating factor	38.9 (50.2±32.4)	21.6 (24.7±13.1)
**GM-CSF**	Granulocyte-Macrophage Colony Stimulating Factor	1.3 (1.5±0.8)	5.2 (4.9±1)
**IFN-α2**	Interferon, alpha2	40.5 (53.6±40.0)	5.7 (5.1±1.8)
**IFN-γ**	Interferon, gamma	3.1 (3.3±1.2)	2.5 (5.5±9)
**IL-10**	Interleukin 10	2.0 (2.3±1.3)	<OOR
**IL-12 (p40)**	Interleukin-12 subunit p40	5.4 (6.5±3.4)	<OOR
**IL-12 (p70)**	Interleukin-12 subunit p70	3.2 (3.3±1.0)	2.1 (2.4±0.7)
**IL-13**	Interleukin 13	23.1 (27.5±16.3)	<OOR
**IL-15**	Interleukin 15	2.3 (2.2±0.5)	<OOR
**IL-17A**	Interleukin 17	<OOR	<OOR
**IL-1RA**	Interleukin-1 receptor antagonist	970.0 (1056.2±687.5)	10.6 (10.3±3.1)
**IL-1α**	Interleukin 1, alpha	16.5 (18.9±7.8)	<OOR
**IL-1β**	Interleukin 1, beta	2.2 (2.4±0.9)	<OOR
**IL-2**	Interleukin 2	1.0 (1.1±0.4)	<OOR
**IL-3**	Interleukin 3	<OOR	<OOR
**IL-4**	Interleukin 4	6.1 (12.6±20.0)	<OOR
**IL-5**	Interleukin 5	1.6 (2.8±3.8)	<OOR
**IL-6**	Interleukin 6	2.7 (2.9±1.6)	<OOR
**IL-7**	Interleukin-7	27.3 (26.8±10.9)	2.5 (2.8±0.9)
**IL-8**	Interleukin 8	19.9 (23.9±8.8)	4.9 (5.9±2.5)
**IP-10**	CXCL10	2891.0 (3413.2±1793.8)	198 (234.7±90.4)
**MCP-1**	CCL2	76.2 (113.9±97.4)	407 (407.8±187)
**MIP-1α**	CCL3	6.3 (7.5±3.9)	0 (2.1±3.2)
**MIP-1β**	CCL4	5.6 (6.3±1.9)	36.9 (42.7±23.5)
**TNF-α**	Tumor necrosis factor, alpha	0.6 (0.5±0.4)	7.6 (7.6±2.9)
**TNF-β**	Tumor necrosis factor, beta	0.0 (0.7±0.8)	<OOR
**VEGF**	Vascular endothelial growth factor	52.6 (55.3±26.1)	79.3 (96±49.7)

[a]Values are expressed as Median (Mean±SD); OOR<: out (below) of detection range

Supplementary Table 2. Identified Networks linking molecules showing kinetic Profile I and Profile II

Profile	Network ID	Molecules in the Network	Score	Focus molecules	Top functions
**I**	1	**MIP-1α**, **MIP-1β**, **CCL11/Eotaxin**, Cpla2, **GM-CSF**, **G-CSF**, **CXCL10/IP10**, Eotaxin, Fcer1, Fcgr3, Ggt, Gm-csf, Gsk3, HLA-DQ, HLA-DR, Ifn gamma, Iga, Ige, **IL-4**, **IL-5**, **IL-10**, IL-15, **IL-12(complex)/IL-12(p70)**, **IL-12(p40)**, Immunoglobulin, Interferon alpha, lymphotoxin-alpha-beta2, MHC Class II (complex), Nr1h, PI3K (complex), STAT5a/b, Tlr, **TNF**, Tnf receptor, U1 snRNP	25	12	Cell-to-cell Signalling and Interaction, Cellular Movement, Immune Cell Trafficking
	2	Akt, Ap1, BCR (complex), CD3, Creb, Cyclin E, **EGF**, ERK, ERK1/2, Hsp27, Hsp90, IFN Beta, **IFN-γ**, IgG, Igm, **IL-2**, **IL-7**, **IL-13**, IL-23, **IL-1α**, **IL-1RA**, JnK, **TNF**, Mapk, Nfat (family), NFkB (complex), P38 MAPK, PI3K (family) Ras, SAA, SRC (family), TCR, Vegf, **VEGF-A**, **VEGF-B**	20	10	Cell-to-cell Signalling and Interaction, Cellular Growth and Proliferation, Inflammatory Response
**II**	3	Ap1, BAI1, **MCP-1**, Cdk, CLEC11A, **IL-8**, DGKH, ECE1, ERK, ERK1/2, Fcer1, FXN, GNA14, Histone h3, IL-1, **IL-6**, IL-36A, Jnk, LDL, LPAR3, MARVELD3, MYO1E, NFkB (complex), NOD1, P38 MAPK, PDGF-BB, PI3K (family), Pkc(s), PLA2G7, STAB2, TFF2, Tlr, TMEM9B, TRIO, VRK2	8	3	Inflammatory Disease, Connective tissue Disorders

Network-eligible molecules/proteins that interact with other molecules in the Ingenuity Knowledge Database.
In bold: focus molecules belonging to the panel of cytokines/chemokines analysed.
